# Investigating Children’s Narrative Abilities in a Chinese and Multilingual Context: Cantonese, Mandarin, Kam and Urdu Adaptations of the Multilingual Assessment Instrument for Narratives (MAIN)

**DOI:** 10.3389/fpsyg.2020.573780

**Published:** 2020-11-20

**Authors:** Rachel T. Y. Kan, Angel Chan, Natalia Gagarina

**Affiliations:** ^1^Department of Chinese and Bilingual Studies, Faculty of Humanities, The Hong Kong Polytechnic University, Kowloon, Hong Kong; ^2^Research Centre for Language, Cognition, and Neuroscience, Department of Chinese and Bilingual Studies, The Hong Kong Polytechnic University, Kowloon, Hong Kong; ^3^Speech Therapy Unit, Department of Chinese and Bilingual Studies, The Hong Kong Polytechnic University, Kowloon, Hong Kong; ^4^PolyU-Research Centre on Chinese Linguistics, The Hong Kong Polytechnic University, Kowloon, Hong Kong; ^5^Leibniz Center for General Linguistics (ZAS), Berlin, Germany

**Keywords:** narrative abilities, language assessment, Developmental Language Disorder, bilingualism/multilingualism, Chinese languages, Urdu, ethnic minority children

## Abstract

This article introduces the LITMUS-MAIN (Language Impairment Testing in Multilingual Settings-MAIN) and motivates the adaptation of this instrument into Chinese languages and language pairs involving a Chinese language, namely Cantonese, Mandarin, Kam, Urdu. We propose that these new adapted protocols not only contribute to the theoretical discussion on story grammar and widen the evidential base of MAIN to include more languages in studying bilinguals, they also offer new methods of assessing language development in young children that have the potential to tease apart the effects of language impairment and bilingualism and improve the identification of Developmental Language Disorder. These new protocols are the first tools to be designed for the dual assessment of language skills in these particular languages, in particular narrative skills in bilingual children speaking these languages. By catering to under-researched languages and over-looked groups of bilingual children, these new tools could improve the clinical management for certain bilingual ethnic minority children such as Urdu-Cantonese and Kam-Mandarin bilinguals, as well as promote the study of these groups and their acquisition issues. Advances in understanding the theoretical and acquisition issues in childhood bilingualism can also be made possible using these new tools.

## Introduction

Narrative tasks provide a window onto a number of key linguistic abilities in a naturalistic setting, including knowledge of lexicon, morphosyntax, and pragmatics (Liles, 1993; Botting, 2002). Narration also involves language use in context, which involves navigating a discourse, other cognitive skills, and even social skills. In addition to oral language and comprehension skills, narrative skills are also important predictors of literacy (e.g. Torrance and Olson, 1984; Norris and Bruning, 1988; McCabe, 1996; Bliss et al., 1998; Griffin et al., 2004; Wallach, 2008). These tasks tap onto multiple domains that are deployed in integration, and are therefore highly useful for assessing language and cognitive development in young children.

MAIN (Multilingual Assessment Instrument for Narratives, Gagarina et al., 2019) as a part of the LITMUS (Language Impairment Testing in Multilingual Settings) battery (Armon-Lotem et al., 2015) was developed during the European Cooperation in Science and Technology (COST) Action IS0804 (2009–2013) *Language Impairment in a Multilingual Society: Linguistic Patterns and the Road to Assessment*. It was designed to assess bilingual development and be sensitive to differences between children with and without Developmental Language Disorder (DLD, e.g., Gopnik and Crago, 1991; Herbert et al., 2004; Bishop et al., 2017). Narratives are less biased against bilinguals, who can generate the basic structure of narratives even in their weaker language, due to the relatively language-independent nature of macrostructure skills (Gutiérrez-Clellen, 2002; Pearson, 2002; Altman et al., 2016), while at the same time differentiating between bilingual children with and without DLD (e.g., Bishop and Donlan, 2005; Gutiérrez-Clellen et al., 2009; Duinmeijer et al., 2012). Moreover, MAIN is cross-linguistically and cross-culturally robust, with more than 200 pictures developed for the task, and has been further modified to suit different cultural contexts in Africa and Asia and to improve the scoring scheme based on empirical evidence.

The development of MAIN began with theoretical conceptualization. Going beyond traditional approaches to narrative assessment that focus mainly on Story Structure, a multi-dimensional model of story organization was elaborated for MAIN, with three dimensions including Story Structure (the number of story components produced), Structural Complexity (complexity of combinations of story components), and Internal State Terms (words and expressions describing the internal or mental state of characters). This led to real-life plots containing elements that are appropriate for children and applicable across different cultures and languages. Pictures depicting these plots were piloted for 15 languages with over 500 children, along with accompanying materials such as standardized procedures for administration, assessment protocols, and scoring guidelines. The published form of MAIN uses four stories with six picture sequences each to elicit the telling’ retelling and telling based upon a model story. Macrostructure elements, which constitute the story grammar and are relevant to the scoring criteria (main episodic elements such as number of goals, attempts, outcomes) are controlled across the four stories (Pearson, 2002). The stories are parallel in content and structure across the two conditions, so MAIN can yield modifiability scores by comparing narrative production in the tell versus retell conditions (Chan et al., 2018). Although this is not a full-fledged dynamic assessment, the degree of modifiability upon modeling can be a measure of learning potential, which is more vulnerable in children with DLD (Peña et al., 2007; Henderson et al., 2018; Orellana et al., 2019). The pictures were also designed to elicit Internal State Terms (showing e.g., emotions, mental states, and perceptual states, e.g., “sad,” “forget,” “hear”) (Miller, 2006; Kobayashi et al., 2008), which targets cognitive linguistic abilities that are restricted in children with DLD. Language-specific microstructure skills can also be evaluated. For each story, comprehension is tested using 10 pre-determined questions based on the picture sequences.

Because of the parallel design across stories, bi/multilingual children can be assessed in each of their languages using a different language version of MAIN. In addition to enabling dual assessment of the two languages in a bilingual child, MAIN incorporates the testing of abilities in both macrostructure and microstructure, which can help differentiate between bilingual children with and without genuine language impairments, as shown in studies such as Altman et al., 2016; Boerma et al., 2016; Tsimpli et al., 2016; Fichman et al., 2017; Fichman and Altman, 2019; Gagarina et al., 2019. There is also a standardized adaptation process (following the guidelines set out in Gagarina et al., 2012, 2019)^1^, so that features at the macrostructural level (e.g., the number and sequence of the story components Goal, Attempt, Outcome, and Internal States for each protagonist) are the same across languages; and features at the microstructural level (e.g., number of coordinating and subordinating constructions, internal state terms overall, number of direct speech sentences) are consistent across the stories and as similar as possible to the English version of MAIN. The pictures remain the same, with minimal cosmetic changes to characters where appropriate. MAIN also targets one of the three main indicators of poor prognosis associated with children with DLD (Bishop et al., 2017), namely scope of problems as manifested in multiple domains, so it is particularly suitable for differentiating between bilinguals with and without DLD. Therefore, MAIN enables the assessment of important skills in young children, and addresses the lack of appropriate developmental expectations and assessment tools for bilingual children. Currently, there are over 70 language versions of MAIN being used in research.

## Motivation for Adapting Main to New Languages

Currently, the languages targeted in acquisition research represent only 1–2% of the world’s languages (Lieven and Stoll, 2013). The bias in child language research is also notable, with 58 and 83% of monolingual and bilingual corpora in the Child Language Data Exchange System (CHILDES) (MacWhinney, 2000) involving languages from the Indo-European family (Stoll, 2015; Kidd, 2017). This implies that literature and tested language acquisition theories have limited applicability across all spoken languages. The clinical basis for assessing language development is similarly focused on certain languages (typically Anglo-European oriented), while other languages lack validated, let alone standardized norm-referenced language assessment instruments (e.g., Urdu, Kam). Even where standardized language assessment instruments exist (e.g., for Cantonese, Mandarin), the conventional norm-referenced approach in diagnosing language impairment often is not appropriate for bilingual children, given that the existing monolingual norms are not applicable for bilinguals and bilingual norms are often not available and very time consuming and costly to establish.

Adapting MAIN to under-investigated languages both research and clinical practice can benefit from, while widening the evidential base of MAIN. Additionally, this will help researchers working on these languages who become part of the international MAIN network. Therefore, four new protocols of MAIN have been adapted by our research team members into Chinese languages and language pairs involving a Chinese language in studying bilinguals, namely Cantonese (Chan et al., 2020; Gagarina et al., 2019), Mandarin (Gagarina et al., 2019; Luo et al., 2020), Kam (Gagarina et al., 2019; Yang et al., 2020), and Urdu (Gagarina et al., 2019; Hamdani et al., 2020). Cantonese and Mandarin are Sinitic languages in the Sino-Tibetan family. Cantonese is a member of the Yue Chinese dialect group and is spoken as the lingua franca in Hong Kong, Macau, and certain places in Guangdong and Guangxi in mainland China. It is also spoken by large communities of ethnic Chinese around the world. Mandarin is the main representative of Sino-Tibetan and Chinese languages, due to the sheer population of its speakers. It is the national language of China, and an official language of Taiwan and Singapore, and also of Hong Kong and Macau under the appellation “Chinese.” It is spoken in overseas Chinese communities, and its second language learners are also increasing in number. Kam is a minority language in the Kam-Tai branch of the Tai-Kadai family^2^, spoken by Kam ethnic minority people mainly in south and southwest China. Finally, Urdu is an Indo-Aryan language that is the national language of Pakistan, and also spoken in India, the United Kingdom, and Canada, etc. Urdu is closely related to Hindi and both languages are mutually comprehensible, but Urdu features significant lexical borrowing from Arabic and Persian.

The choice of these target languages is grounded in a number of reasons, firstly dealing with the lack of assessment materials and research on certain languages with a large number of speakers, which reciprocally also adds typological diversity to MAIN, in particular in terms of languages paired with a Chinese language in bilingual acquisition. Mandarin and Urdu are among the most-spoken languages in the world, ranking first and 20th in terms of the number of first language speakers (918 and 70 million respectively, Eberhard et al., 2019; Urdu Language, 2019). In addition, they have a further 199 and 102 million second language speakers respectively. As for Cantonese, there are more than 73 million first language speakers, including more than 6 million using it as their daily language in Hong Kong (89% of population, Census and Statistics Department, Government of the Hong Kong Special Administrative Region, 2018). The population of Kam-speaking people is at about 2.9 million (National Bureau of Statistics, People’s Republic of China, 2019).

The addition of Cantonese, Mandarin, Kam, and Urdu widen the evidential base of MAIN to include more typologically diverse languages. There had been no Sino-Tibetan or Tai-Kadai language versions of MAIN until Cantonese, Mandarin, and Kam, and Urdu is one of the first Indo-Aryan adaptations of MAIN. These new language families introduce new structures and features that will be covered by MAIN’s assessment protocols. For example, Cantonese, Mandarin, and Kam are isolating languages. Other typological features that are particularly relevant to narratives include, for instance, topic-prominence and argument ellipsis, and the use of bare nominals to introduce new referents. Urdu uses postpositions, and does not have articles. These features contrast with the properties of most languages currently under investigation in similar initiatives (typically European languages). The canonical word order (Subject-Object-Verb) of Urdu is also less frequent among the languages currently covered by MAIN, occurring only in Turkish (published) and Kurmanji (in development) (excluding languages that allow for SOV structures in certain contexts), even though SOV languages make up more than 40% of languages in the world (Hammarström, 2016). (The canonical word order for Cantonese, Mandarin, and Kam is Subject-Verb-Object, which occurs more widely in other MAIN languages).

Given that quantitative and qualitative evaluation of narrative skills can be assessed via macrostructure and microstructure, analyses of these new typologically diverse languages might open new perspectives on the developmental trajectories of narration in children (and also on narrative skills in adults). Data collected on these four new languages using MAIN can reveal whether aspects of narration that were previously found vulnerable to impairment (e.g., use of internal state terms and referencing expressions) are similarly affected in other language families, as well as whether there are any effects of impairment on properties that are more unique to these new languages, thereby extending previous work to bilingual speakers in South/East Asia.

Social concerns were also a motivation in the selection of these new target languages. The inclusion of Kam and Mandarin enables the study of children from the Kam ethnic minority, who grow up in a unique social-communicative environment in mainland China. Some Kam people have a low socio-economic status, as they come from areas such as Guangxi, which is an underdeveloped province in China (National Bureau of Statistics, People’s Republic of China, 2019). Many Kam-speaking children become “left-behind” as they remain in rural, even remote areas while their parent(s) move to cities seeking employment. They are among 7 million “left-behind” children across China (Ministry of Civil Affairs, People’s Republic of China, 2018). These children are primarily taken care of by their grandparent(s) (96% of these children) or other relatives/family friends (4%), who often have low education levels, do not interact with children in the same way as parents would, and provide insufficient support for the children’s language development. In addition, Mandarin is the national language of Mainland China, and consequently Kam ethnic minority children grow up bilingually, with Kam (the minority language) acquired as the first and family language, and Mandarin as the second and school language. Since Kam-speaking children grow up in communication environments non-conducive for children’s early language development, an important social concern is the negative developmental outcomes in these children, such as the occurrence of behavioral and psychological problems in these children (see Wang and Mesman, 2015 for a review). However, the impact of such a developmental environment on their language abilities has received little attention, with important questions such as whether the reduced input condition in both languages would lead to incomplete acquisition in either or both languages. Therefore, assessment tools that are suitable for conducting cross-linguistic/cross-cultural comparisons are needed, in order to document the language outcomes and learner characteristics of these children and allow comparisons with age peers in other linguistic or cultural contexts. MAIN would be an excellent tool for conducting these comparisons. In addition, bilingual children face a problem of under- or over-identification of language impairments, and MAIN has the potential to differentiate bilingual children with and without DLD, and to better specify the kind of social services needed (i.e., second language support or direct clinical intervention). Similarly, the Mandarin protocol has the potential to help address social issues for the many other Mandarin-speaking bilingual children, such as ethnic minorities in China and heritage speakers in countries with other societal languages.

The inclusion of Cantonese and Urdu were also similarly motivated i.a. by social concern for Urdu-speaking bilingual children in countries where it is not a main/majority language, and the lack of tools suitable for assessing children speaking these languages. In Hong Kong, Urdu is spoken by Pakistanis (among others), who make up around 16% (13,492) of South Asian people in Hong Kong (Census and Statistics Department, Government of the Hong Kong Special Administrative Region, 2017). Most of them (>80%) do not use the official languages of Hong Kong or any of the Chinese languages as their usual language, so children speaking Urdu at home can only acquire Cantonese, the lingua franca, in the limited context of school or society, as a second or additional language. At the same time, they receive Urdu input at home only, and not in society or school due to the relatively small number of speakers. Many students in Hong Kong speaking a language other than Cantonese at home report difficulties in learning and using Cantonese (e.g., Shum et al., 2011), but the development of Cantonese in bilingual ethnic minority children is vastly understudied (see Cheung (2017) as a notable exception in recent years). Their Cantonese development in this particular linguistic environment or the effects on academic outcomes is poorly understood, and little is available in terms of language developmental expectations and appropriate language assessment tools (Yao et al., 2019; Chan et al., 2020). Therefore, MAIN can help document the development of language abilities in both Urdu and Cantonese to inform the developmental expectations for these children. In addition, existing Cantonese standardized tests are all normed on predominantly monolingual children, and to date, there are no standardized tests to assess Urdu-speaking children (not even for monolinguals), so adapting MAIN to Cantonese and Urdu will provide the first tools for practitioners to assess the languages skills of children with Urdu as family or additional language in Hong Kong, and even potentially children speaking Urdu as the majority language (in Pakistan), although it has yet to be norm-referenced.

Additionally, Urdu MAIN can be paired with other language versions to assess bilinguals speaking Urdu as a minority language in other countries, such as in the United Kingdom, where there are 0.4 million first language speakers of Urdu (0.61% of population, Eberhard et al., 2019) or in the United States, where there are 0.40 million people using Urdu at home (0.14% of population, U.S. Census Bureau, 2015). Cantonese MAIN can also be appropriate for assessing heritage speakers of Cantonese growing up in countries with a different majority language, such as the United States, where there are 0.46 million people speaking Cantonese at home (0.16% of population, U.S. Census Bureau, 2015), Canada, where 0.57 million people with Cantonese as mother tongue (1.3% of population, Statistics Canada/Statistique Canada, 2016), or Australia where there are 0.28 million people speaking Cantonese at home (1.2% of population, Australian Bureau of Statistics, 2017). In sum, the four newly-added protocols for MAIN will help address concerns for significant bilingual demographic groups who tend to receive little support in terms of language development and whose language(s) cannot be readily evaluated using other existing assessment tools. Last, but not least, semi-spontaneous data on these four languages in a heritage context will provide an empirical base for theoretical research targeting the restructuring of heritage grammars and for new developments in language change statistical modeling.

The protocols and accompanying introductory articles can be found here: https://zaspil.leibniz-zas.de/issue/view/55.

## Discussion

Adapting MAIN to Cantonese, Mandarin, Urdu, and Kam has a number of important implications in clinical, social, theoretical linguistics and other areas as well as language documentation. First, it will be used in clinical practice and has the potential to improve assessment, support, and therapy services for bilingual children across different communities and countries. Speech-language therapists and educators need to take multilingualism and multiculturalism into account in their professional practice, as these bilingual children – especially ethnic minorities – are increasingly seen on their caseloads in recent years. If there are measures assessed by MAIN that have been demonstrated to be sensitive to language impairment in these languages, MAIN could be directly considered for clinical use. In the least, in cases when local speech and language therapists in certain countries are not familiar with one of a bilingual child’s languages that has a minority status (so dual language testing is not always possible), language tests suitable for such children allows us to generate some reference or even norming data to formulate appropriate developmental expectations for these children.

In an academic context, these tools are part of an initiative to document the language abilities of ethnic minorities in different countries, such as South Asian bilingual minority children in Hong Kong and mainland China, and heritage speakers of Cantonese and Mandarin in English-speaking countries. These new protocols lay the groundwork for further studies of clinical populations speaking these target languages, and these instruments can also be used more generally as inclusionary/exclusionary measures for academic research or for language profiling. For example, the Urdu and Cantonese versions of MAIN have been used to assess bilingual children with and without suspected language impairments, and show promise to differentiate between the two groups (Chan et al., 2018; Chan et al., under revision). Another example of using MAIN in research is Sheng et al. (2020), where there is evidence for differentiation between Mandarin-speaking children at risk for DLD and typically developing controls using Mandarin MAIN.

More generally, a range of acquisition issues can also be studied using MAIN. It allows one or more languages in the same child to be assessed, in different elicitation modes (including story telling, story retelling, model story, and comprehension). This enables extensive comparisons between bilingual children’s dual languages at both macrostructure and microstructure levels, and their interfaces. These tools can also promote the study of certain bilingual ethnic minority children such as Urdu-Cantonese and Kam-Mandarin bilinguals and their acquisition issues.

There are also theoretical contributions to be made through studying Cantonese, Mandarin, Urdu, and Kam, as findings based on these languages could bear on the theoretical themes of universals and variations in language acquisition, and also allow theories accounting for phenomena such as language attrition or heritage language restructuring and input effects to be tested. These languages also add typological diversity to MAIN, and in many cases they themselves are under-documented in the literature, especially for Urdu and Kam.

## Conclusion

Multilingual Assessment Instrument for Narratives can be used to assess competence in narrative comprehension and production in bilingual (and monolingual) children. Adding Cantonese, Mandarin, Urdu, and Kam strengthens MAIN’s mission to enable parallel assessment of a bilingual’s narrative abilities in two languages and broadens the MAIN network of researchers and practitioners. MAIN is designed to assess multiple facets of language, and be insensitive to effects of bilingualism but sensitive to language impairment, which is an advantage over other standardized tests with monolingual norms. These new protocols have the potential to allow for differentiation between bilingual children who are typically developing but diverging from monolingual peers due to bilingualism, and those with genuine language impairment. Accordingly, the appropriate intervention or support could be provided, and clinical management steered more precisely.

The assessment protocols and accompanying introductory articles for all four languages are available through open access to all researchers and speech-therapy practitioners (citations above). This versatile tool can be easily incorporated into clinical linguistics studies and other research involving the narrative abilities of monolingual and bilingual children. Further validation is required for these new versions of MAIN, such as their clinical accuracy in differentiating children with and without language impairment, but meanwhile, these tools and data gathered using them will form the foundation for less biased assessment of language acquisition in bilingual children as well as the identification of language impairments. It is also hoped that creating these new resources that are suitable for overlooked or disadvantaged children will promote equal access to support for learning and development, and highlight the issues faced by such children.

## Data Availability Statement

Publicly available datasets were analyzed in this study. This data can be found here: The four adapted assessment protocols can be downloaded after registration here: https://www.leibniz- zas.de/index.php?id=964. The accompanying introductory articles for these new protocols are published in Issue 64 of ZAS Paper in Linguistics (ZASPiL): https://zaspil.leibniz- zas.de/article/view/553 (Cantonese), https://zaspil.leibniz- zas.de/article/view/569 (Mandarin), https://zaspil.leibniz-zas.de/article/view/567 (Kam), https://zaspil.leibniz-zas.de/article/view/580 (Urdu).

## Author Contributions

RK drafted the manuscript and is an author of three of the tools discussed in this manuscript. AC conceptualized this manuscript and led the initiatives to adapt MAIN into Cantonese, Mandarin, Kam and Urdu with her research team, and provided edits on the manuscript. NG and her research team created MAIN in 2002 and since then has been supporting and promoting the adaptation of MAIN to new languages on a global scale and supported AC and RK and members in their research team to adapt MAIN into Cantonese, Mandarin, Kam and Urdu. All authors approved the final version of the manuscript.

## Conflict of Interest

The authors declare that the research was conducted in the absence of any commercial or financial relationships that could be construed as a potential conflict of interest.

## Footnotes

1 An adjusted version of the guidelines can be viewed in Bohnacker and Gagarina (2020) here: https://doi.org/10.21248/zaspil.64.2020.549.

2 The genetic relationship of Kam-Tai languages is still under debate with some arguing they are Sino-Tibetan languages (Li, 1965, 1973) and others arguing they are Austronesian languages (Benedict, 1942, 1975, 1990).

## References

[B1] Altman C. Armon-Lotem S. Fichman S. Walters J. (2016). Macrostructure, microstructure, and mental state terms in the narratives of English–Hebrew bilingual preschool children with and without specific language impairment. *Appl. Psycholinguist.* 37:1 10.1017/S0142716415000466

[B2] Armon-Lotem S. de Jong J. Meir N. (eds) (2015). *Assessing Multilingual Children: Disentangling Bilingualism from Language Impairment.* Bristol: Multilingual Matters.

[B3] Australian Bureau of Statistics (2017). *Census of Population and Housing: Reflecting Australia - Stories from the Census, 2016.* Canberra: Australian Bureau of Statistics.

[B4] Benedict P. K. (1942). Thai, Kadai, and Indonesian: a new alignment in Southeastern Asia. *Am. Anthropol.* 44:4. 10.1525/aa.1942.44.4.02a00040 33021500

[B5] Benedict P. K. (1975). *Austro-Thai Language and Culture with a Glossary of Roots.* New Haven: Human Relations Area Files Press.

[B6] Benedict P. K. (1990). *Japanese/Austro-Tai.* Ann Arbor: Karoma.

[B7] Bishop D. Donlan C. (2005). The role of syntax in encoding and recall of pictorial narratives: evidence from specific language impairment. *Br. J. Dev. Psychol.* 23:1. 10.1348/026151004X20685 30467716

[B8] Bishop D. V. M. Snowling M. J. Thompson P. A. Greenhalgh T. Adams C. Archibald L. (2017). Phase 2 of CATALISE: a multinational and multidisciplinary Delphi consensus study of problems with language development: terminology. *J. Child Psychol. Psychiatry Allied Disciplines* 58:10. 10.1111/jcpp.12721 28369935 PMC5638113

[B9] Bliss L. S. McCabe A. Miranda E. A. (1998). Narrative assessment profile: discourse analysis for school-age children. *Journal of Communication Disorders*, 31 347–363. 10.1016/S0021-9924(98)00009-4 9697044

[B10] Boerma T. Leseman P. Timmermeister M. Wijnen F. Blom E. (2016). Narrative abilities of monolingual and bilingual children with and without language impairment: implications for clinical practice. *Int. J. Lang. Commun. Disord.* 51 626–638. 10.1111/1460-6984.12234 26989878

[B11] Bohnacker U. Gagarina N. (2020). Introduction to MAIN–Revised, how to use the instrument and adapt it to further languages. *ZAS Papers Linguist.* 64 13–21.

[B12] Botting N. (2002). Narrative as a tool for the assessment of linguistic and pragmatic impairments. *Child Lang. Teach. Ther.* 18 1–21. 10.1191/0265659002ct224oa

[B13] Census and Statistics Department, Government of the Hong Kong Special Administrative Region (2017). *Hong Kong 2016 Population By-census - Thematic Report: Ethnic Minorities.* Hong Kong: Census and Statistics Department.

[B14] Census, and Statistics Department, Government of the Hong Kong Special Administrative Region (2018). *Main table A107, 2016 Population By-census.* Hong Kong: Census and Statistics Department.

[B15] Chan A. Chen S. Tse B. Hamdani S. Cheng K. (under revision) Story telling in bilingual Urdu-Cantonese ethnic minority children in Hong Kong: macrostructure and its relation to linguistic skills. *Linguist. Approach. Bilingualism.* 10.3389/fpsyg.2023.924056 PMC10009217 36923152

[B16] Chan A. Cheng K. Kan R. Wong A. M.-Y. Fung R. Wong J. (2020). The Multilingual Assessment Instrument for Narratives (MAIN): Adding Cantonese to MAIN. *ZAS Papers Linguist.* 64 23–29.

[B17] Chan A. Chui B. Lo J. Luk P. Gagarina N. (2018). “Narrative abilities of bilingual Urdu-Cantonese ethnic minority children in Hong Kong,” in *Poster presented at Child Language Symposium*, Reading.

[B18] Cheung H. T. (2017). “L2 oral narrative development in ethnic minority children of Hong Kong,” in *Poster presented at the IASCL congress*, Lyon France.

[B19] Duinmeijer I. de Jong J. Scheper A. (2012). Narrative abilities, memory and attention in children with a specific language impairment. *Int. J. Lang. Commun. Disord.* 47:5. 10.1111/j.1460-6984.2012.00164.x 22938065

[B20] Eberhard D. M. Simons G. F. Fennig C. D. (eds) (2019). *Ethnologue: Languages of the World*, 22nd Edn Dallas: SIL International.

[B21] Fichman S. Altman C. (2019). Referential cohesion in the narratives of bilingual and monolingual children with typically developing language and with specific language impairment. *J. Speech Lang. Hear Res.* 62 1–20. 30950755 10.1044/2018_JSLHR-L-18-0054

[B22] Fichman S. Altman C. Voloskovich A. Armon-Lotem S. Walters J. (2017). Story grammar elements and causal relations in the narratives of Russian-Hebrew bilingual children with SLI and typical language development. *J. Commun. Disord.* 69 72–93. 10.1016/j.jcomdis.2017.08.001 28886430

[B23] Gagarina N. Klop D. Kunnari S. Tantele K. Välimaa T. Bohnacker U. (2019). MAIN: Multilingual Assessment Instrument for Narratives – Revised. *ZAS Papers in Linguistics* 63 Berlin: ZAS.

[B24] Gagarina N. Klop D. Kunnari S. Tantele K. Välimaa T. Balčiūnienė I. (2012). *MAIN: Multilingual Assessment Instrument for Narratives. ZAS Papers in Linguistics (ZASPiL)*, 56 Berlin: ZAS.

[B25] Gopnik M. Crago M. B. (1991). Familial aggregation of a developmental language disorder. *Cognition* 39:1 10.1016/0010-0277(91)90058-C 1934976

[B26] Griffin T. M. Hemphill L. Camp L. Wolf D. P. (2004). Oral discourse in the preschool years and later literacy skills. *First Language*, 24 123–147. 10.1177/0142723704042369

[B27] Gutiérrez-Clellen V. F. (2002). Narratives in two languages: assessing performance of bilingual children. *Linguist. Educ.* 13:2 10.1016/S0898-5898(01)00061-4

[B28] Gutiérrez-Clellen V. F. Simon-Cereijido G. Erickson Leone A. (2009). Code-switching in bilingual children with specific language impairment. *Int. J. Bilingualism* 13:1. 10.1177/1367006909103530 22611333 PMC3354921

[B29] Hamdani S. Z. Kan R. Chan A. Gagarina N. (2020). *The Multilingual Assessment Instrument for Narratives (MAIN): Adding Urdu to MAIN. ZAS Papers in Linguistics (ZASPiL)* 64 Berlin: ZAS.

[B30] Hammarström H. (2016). Linguistic diversity and language evolution. *Journal of Language Evolution.* 1 1 10.1093/jole/lzw002

[B31] Henderson D. E. Restrepo M. A. Aiken L. S. (2018). Dynamic assessment of narratives among navajo preschoolers. *J. Speech Lang. Hear. Res.* 61:10 10.1044/2018_JSLHR-L-17-0313 30304364

[B32] Herbert M. R. Ziegler D. A. Makris N. Filipek P. A. Kemper T. L. Normandin J. J. (2004). Localization of white matter volume increase in autism and developmental language disorder. *Ann. Neurol.* 55:4. 10.1002/ana.20032 15048892

[B33] Kidd E. (2017). “Widening the contexts of language acquisition,” in *Proceedings of the Presented at the 2nd International Conference on Documentary Linguistics – Asian Perspectives: Transformations and sustainability* (Hong Kong: The University of Hong Kong).

[B34] Kobayashi C. Glover G. H. Temple E. (2008). Switching language switches mind: linguistics effects on development neural bases of ‘Theory of Mind’. *Soc. Cogn. Affect. Neurosci.* 3:1. 10.1093/scan/nsm039 19015096 PMC2569814

[B35] Li F. K. (1965). The Tai and the Kam-Sui languages. *Lingua* 14 5–6. 10.1016/0024-3841(65)90040-9

[B36] Li F. K. (1973). Languages and dialects of China. *J. Chinese Linguist.* 1:1.

[B37] Lieven E. Stoll S. (2013). Early communicative development in two cultures: a comparison of the communicative environments of children from two cultures. *Hum. Dev.* 56:3 10.1159/000351073

[B38] Liles B. Z. (1993). Narrative discourse in children with language disorders and children with nor- mal language: a critical review of the literature. *J. Speech Lang. Hear Res.* 36:5. 10.1044/jshr.3605.868 8246476

[B39] Luo J. Yang W. C. Chan A. Cheng K. Kan R. Gagarina N. (2020). *The Multilingual Assessment Instrument for Narratives (MAIN): Adding Mandarin to MAIN. ZAS Papers in Linguistics (ZASPiL)* 64 Berlin: ZAS.

[B40] MacWhinney B. (2000). *The CHILDES Project: Tools for analyzing talk (Third Edition).* Hillsdale, NJ: Lawrence Erlbaum Associates.

[B41] McCabe A. (1996). “Evaluating narrative discourse skills,” in *Assessment of Communication and Language*, eds Cole K. Dale P. Thal D. (Baltimore, MD: Paul H. Brookes), 121–142.

[B42] Miller C. A. (2006). Developmental relationships between language and theory of mind. *Am. J. Speech Lang. Pathol.* 41:5. 10.1044/1058-0360(2006/014) 16782686

[B43] Ministry of Civil Affairs, People’s Republic of China (2018). *Figures: Rural left-behind children data 2018.* Beijing: Ministry of Civil Affairs, People’s Republic of China.

[B44] National Bureau of Statistics, People’s Republic of China (2019). *China statistical yearbook.* Beijing: China Statistical Press.

[B45] Norris J. A. Bruning R. H. (1988). Cohesion in the narratives of good and poor readers. *Journal of Speech and Hearing Disorders*, 53 416–424. 10.1044/jshd.5304.416 3184902

[B46] Orellana C. I. Wada R. Gillam R. B. (2019). The use of dynamic assessment for the diagnosis of language disorders in bilingual children: a meta-analysis. *Am. J. Speech Lang. Pathol.* 28:3 10.1044/2019_AJSLP-18-0202 31194570

[B47] Pearson B. Z. (2002). “Language and mind in the stories of bilingual children,” in *Narrative Development in a Multilingual Context*, eds Verhoeven L. Stromqvist S. (Amsterdam: John Benjamins), 135–174.

[B48] Peña E. D. Reséndiz M. Gillam R. B. (2007). The role of clinical judgements of modifiability in the diagnosis of language impairment. *Adv. Speech Lang. Pathol.* 9 332–345. 10.1080/14417040701413738

[B49] Sheng L. Shi H. Wang D. Hao Y. Zheng L. (2020). Narrative production in Mandarin-speaking children: Effects of language ability and elicitation method. *J. Speech Lang. Hear Res.* 63:3 10.1044/2019_JSLHR-19-00087 32163319

[B50] Shum M. S. Gao F. Tsung L. Ki W. W. (2011). South Asian students’ Chinese language learning in Hong Kong: motivations and strategies. *J. Multilingual Multicult. Dev.* 32 285–297. 10.1080/01434632.2010.539693

[B51] Statistics Canada/Statistique Canada (2016). *2016 Census of Population.* Ottawa, ON: Statistics Canada/Statistique Canada.

[B52] Stoll S. (2015). “Studying language acquisition in different linguistic and cultural settings,” in *The Routledge Handbook of Linguistic Anthropology*, ed. Bonvillain N. (New York, NY: Routledge), 140–158.

[B53] Torrance N. Olson D. R. (1984). “Oral language competence and the acquisition of literacy,” in *The Development of Oral and Written Language in Social Contexts*, ed. Pellegrini A. Yawkey T. (Norwood, NJ: Ablex), 167–181.

[B54] Tsimpli I. Peristeri E. Andreou M. (2016). Narrative production in monolingual and bilingual children with specific language impairment. *Appl. Psycholinguist.* 37 195–216. 10.1017/s0142716415000478

[B55] U.S. Census Bureau (2015). *American Community Survey, Table 1. Detailed Languages Spoken at Home and Ability to Speak English for the Population 5 Years and Over for United States: 2009-2013.* Suitland, MD: U.S. Census Bureau.

[B56] Urdu Language (2019). *In Encyclopaedia Britannica Online.* Available online at: https://www.britannica.com/topic/Urdu-language (accessed May 27, 2020).

[B57] Wallach G. P. (2008). *Language Intervention for School-age Students: Setting Goals for Academic Success.* St. Louis, MO: Mosby.

[B58] Wang L. Mesman J. (2015). Child development in the face of rural-to-urban migration in China: a meta-analytic review. *Perspect. Psychol. Sci.* 10 813–831. 10.1177/1745691615600145 26581737

[B59] Westby C. E. (2005). “Assessing and facilitating text comprehension problems,” in *Language and Reading Disabilities*, eds Catts H. Kamhi A. (Boston: Allyn & Bacon), 157–232.

[B60] Yang W. C. Chan A. Gagarina N. (2020). *The Multilingual Assessment Instrument for Narratives (MAIN): Adding Kam to MAIN. ZAS Papers in Linguistics (ZASPiL) 64.* Berlin: ZAS.

[B61] Yao Y. Chan A. Fung R. Wu W. L. Leung N. Lee S. (2019). Cantonese tone production in pre-school Urdu–Cantonese bilingual minority children. *Int. J. Bilingual.* 24, 767–782. 10.1177/1367006919884659

